# lncRNA USP30-AS1 sponges miR-765 and modulates the progression of colon cancer

**DOI:** 10.1186/s12957-022-02529-x

**Published:** 2022-03-08

**Authors:** Chengren Li, Xu Liang, Yongguang Liu

**Affiliations:** Department of Anorectal Surgery, Weifang People’s Hospital, No.151, Guangwen Street, Weifang, 261000 Shandong China

**Keywords:** lncRNA USP30-AS1, Colon cancer, miR-765, Prognosis, Tumor progression, Cellular processes

## Abstract

**Background:**

The incidence and mortality of colon cancer is increasing recently. It is necessary to identify effective biomarkers for the progression and prognosis of colon cancer. To assess the potential of lncRNA USP30-AS1 (USP30-AS1) in serving as the biomarker of colon cancer and unearth the underlying mechanism.

**Methods:**

There were 123 colon cancer patients enrolled. The expression of USP30-AS1 was evaluated with PCR in tissue and cell samples. The clinical significance of USP30-AS1 was assessed with a series of statistical methods, while the CCK8 and Transwell assay were conducted to estimate its biological effect on the colon cancer cellular processes. In mechanism, the interaction of USP30-AS1 with miR-765 was evaluated with the dual-luciferase reporter assay.

**Results:**

In colon cancer tissues, the USP30-AS1 downregulation and the miR-765 upregulation were observed, and there was a negative correlation between the USP30-AS1 expression level and the miR-765 expression level. The downregulation of USP30-AS1 related to the malignant progression and served as an adverse prognostic indicator of colon cancer. The overexpression of USP30-AS1 dramatically suppressed colon cancer cellular processes, which was alleviated by miR-765.

**Conclusions:**

USP30-AS1 predicts the malignancy and prognosis of colon cancer patients. USP30-AS1 suppressed the progression of colon cancer through modulating miR-765.

## Introduction

Colon cancer is a malignant tumor in the digestive system, which usually occurs at the junction of the rectum and sigmoid colon [1]. Colon cancer is one of the major reasons responsible for cancer-related death in China and also ranked a top position in cancer incidence worldwide. With the changes in the daily diets, the incidence and mortality increased rapidly [2]. Due to the heterogeneity, there is a large deviation in the prediction of disease development only based on the clinical stage of patients. Although great progress has been made in the clinical management of colon cancer, the local recurrence and distant metastasis are still commonly occurred after surgery, which resulted in the poor prognosis of patients [3]. Therefore, the exploration of novel biomarkers that predict colon cancer prognosis and progression could help formulate a better therapeutic strategy.

Long non-coding RNAs (lncRNAs) are a series of vital members in the ncRNA family with a length of over 200 nucleotides and structures similar to mRNAs, which can suppress or induce the coding of neighboring genes [4]. In the past decades, the regulatory effect of lncRNAs, especially the differently expressed lncRNAs in the onset and development of malignant tumors has been revealed [5–7]. Recent studies focused on the lncRNA expression profile in colon cancer have dug out a number of hub genes with abnormal expression and great potential of regulating tumor progression. Xu et al. established a hierarchical cluster profile of aberrantly expressed lncRNAs in colon cancer with the data from the TCGA database and identified a number of potential functional lncRNAs, including lncRNA USP30-AS1 (USP30-AS1) [8]. USP30-AS1 is an antisense lncRNA that locates on chr12 (q24,11), the opposite strand of USP30 locus. Previously, USP30-AS1 has been reported to mediate the progression of various human diseases, such as glioblastoma, cervical cancer, and acute myeloid leukemia [9–11]. However, the specific dysregulation and the function of USP30-AS1 in colon cancer remains unknown, which might be a biomarker of the tumor progression and an indicator of clinical prognosis.

In mechanism, sponging microRNAs (miRNAs) that play roles in cancer development is the main mechanism that mediates the function of lncRNAs [12]. Therefore, the correlated function miRNAs involved in t USP30-AS1’s function in colon cancer progression are also of great significance to understand the potential of USP30-AS1 in serving biomarkers of colon cancer.

## Materials and methods

### Study subjects

This study had been approved by the Ethics Committee of Weifang People’s Hospital. A total of 123 colon cancer patients were enrolled according to the following criteria: (1) the patients primarily diagnosed with colon cancer at Weifang People’s Hospital and were suitable to receive the surgical resection; (2) the patients had never received any anti-tumor therapies, novel adjuvant chemotherapy, or target therapies before the surgery; (3) the patients had never suffered from other malignant tumors; and (4) the clinical records of the patients are completed, and the patients have signed the informed consent. The patients with non-primary colon cancer were excluded.

### Tissue samples and cell culture

Tumor tissues and normal paracancer tissues (at least 5 cm from the lesion) were collected during the surgery and stored in liquid nitrogen. All patients were followed up for 5 years on the telephone to monitor their development.

Colon cancer cells (COLO320, SW480, RKO, and HCT116 cells) and a normal cell were obtained from ATCC and maintained in the RPMI-1640 culture medium with FBS (10%) 37°C and 5% CO_2_.

### USP30-AS1 and miR-765 expression evaluation

Tissue samples and cells were digested with trypsin for the isolation of total RNA with Trizol reagent. After the evaluation of RNA concentration and purity, the extracted RNA was reversed to cDNA with the PrimeScript RT reagent Kit (Takara, Japan) for USP30-AS1 and the TaqMan miR Reverse Transcriptase Kit (Applied Biosystems, USA) for miR-765. The real-time quantitative PCR was performed with the SYBR kit and the CFX96 system (Bio-Rad), and the expression levels were calculated with the 2^−ΔΔCT^ method. The primer sequences were as follows: USP30-AS1 forward 5’-AGCAATAGCTGACGGACCAC-3’, reverse 5’-TGAAAACCAAGCAGCCCCA-3’, miR-765 forward 5’-TGGAGGAGAAGGAAGGTG-3’, and reverse 5’-GAACATGTCTGCGTATCTC-3’. While the reaction conditions of PCR were predenaturation at 94°C for 5 min followed by 40 cycles of 15 s at 94°C, and then, 60°C for 30 s.

### Cell transfection

The pcDNA 3.1-USP30-AS1 was transfected into the colon cancer cells to overexpress USP30-AS1, while miR-765 was silenced by miR-765 inhibitor (5’-CAUCACCUUCCUUCUCCUCCA-3’) and overexpressed by miR-765 mimic (5’-UGGAGGAGAAGGAAGGUGAUG-3’) with the Lipofectamine 2000 (Invitrogen, USA). The transfection efficiency was assessed by their corresponding expression levels.

### Dual-luciferase reporter assay

The wild-type reporter plasmid of USP30-AS1 was synthesized by cloning the fragments containing the miR-765 binding sites. While the mutant-type plasmid was established with the fragments containing the mutant sites. The constructed plasmids and miR-765 mimic or inhibitor were with co-transfected into colon cancer cells with the Lipofectamine 2000 (Invitrogen, USA). After incubation of 48 h, the dual-luciferase reporter assay system (Promega, USA) was conducted to assess the intensity of USP30-AS1 with Renilla as the internal reference.

### Cell proliferation assay

Cells were grown in the 96-well plates and maintained with the completed culture medium for 0, 1, 2, and 3 days at the cell culture condition. Then, the CCK8 was added to each well and incubated for a further 2 h. The viability of cultured cells was evaluated by the absorbance at 450 nm.

### Cell metastasis assay

Cells were seeded on the uncoated (for migration) or Matrigel-coated (for invasion) upper chambers of the 24-well Transwell plates. The upper chambers were filled with the RPMI-1640 medium without FBS, and the bottom chamber was full of FBS-containing RPMI-1640 medium. The plates were incubated at 37°C for 24 h followed by removing the cells on the upper surface. The metastasis cells were fixed with 4% paraformaldehyde and stained with crystal violet before viewed under a microscope. Five random fields were selected, and the average cell number was calculated.

### Statistical analysis

All experiments were performed in triplicate with three independent determinations. All data were expressed as mean ± SD. and analyzed with SPSS 20.0 software. The differences were evaluated by Student’s *t* test or one-way ANOVA followed by the Turkey post hoc test. The chi-square test was performed to evaluate the value of USP30-AS1 in colon cancer progression. While the Kaplan-Meier and Cox regression analyses were performed to assess the prognostic value of USP30-AS1. The statistical significance was indicated by *P* < 0.05.

## Results

### The expression of USP30-AS1 and miR-765 in tissues samples

**Fig. 1:**
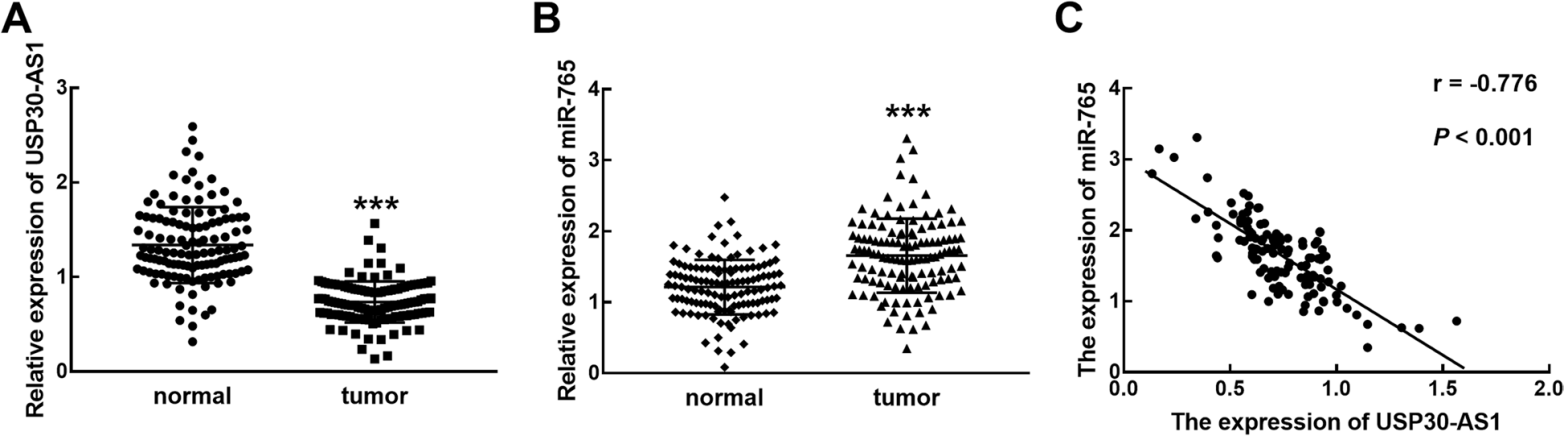
The expression of USP30-AS1 and miR-765 in colon cancer tissues and their correlation. **A**, **B** The downregulation of USP30-AS1 (**A**) and the upregulation of miR-765 (**B**) were observed in colon cancer tissues compared with normal tissues. **C** The expression of USP30-AS1 was negatively correlated with the expression of miR-765 with a correlation coefficient of −0.776, ***P < 0.001

In collected tissues, a significantly reduced expression of USP30-AS1 was found in colon cancer tissues relative to the normal tissues (*P* < 0.001, Fig. 1A). On the contrary, miR-765 was observed to significantly upregulate in colon cancer tissues compared with the normal tissues (*P* < 0.001, Fig. 1B). Notably, a significant negative correlation was found between the expression levels of USP30-AS1 and miR-765 with a correlation coefficient of −0.776 (*P* < 0.001, Fig. 1C).

### The association of USP30-AS1 with the clinical features of patients

**Fig. 2:**
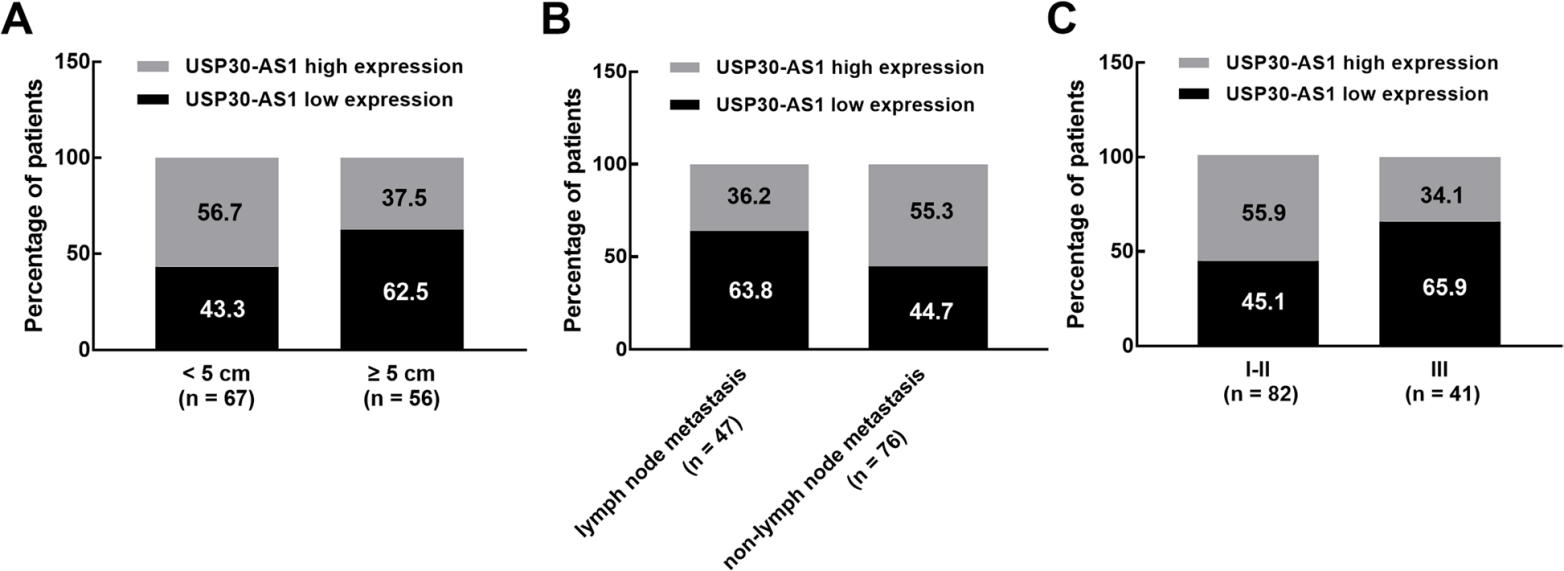
The proportion of low USP30-AS1 expression and high USP30-AS1 expression in patients with various tumor sizes (**A**), lymph node metastasis status (**B**), and TNM stage (**C**)

According to the average expression level of USP30-AS1 in colon cancer tissues, the enrolled patients were grouped as the low USP30-AS1 group with 64 patients and the high USP30-AS1 group with 59 patients. It was found that a big proportion of patients with a relatively larger tumor size showed a low expression of USP30-AS1 (62.5 vs. 37.5%, Fig. 2A). Similarly, a larger percentage of lower USP30-AS1 was also observed in the patients with lymph node metastasis (63.8 vs. 36.2%, Fig. 2B) and advanced TNM stage (65.9 vs*.* 34.1%, Fig. 2C). Consistently, USP30-AS1was demonstrated to closely related to patients’ tumor size (*P* = 0.034), lymph node metastasis (*P* = 0.039), and TNM stage (*P* = 0.030, Table 1).

**Table 1:** Association between USP30-AS1 and the clinicopathological features of patients

	Cases (*n* = 123)	USP30-AS1 expression		*P*
		Low	High	
Age				0.789
< 50	62	33	29	
≥ 50	61	31	30	
Sex				0.736
Male	79	42	37	
Female	44	22	22	
Tumor size (cm)				0.034
<5	67	29	38	
≥ 5	56	35	21	
Grade of differentiation				0.160
High + moderate	82	39	43	
Poor	41	25	16	
Lymph node metastasis				0.039
With	47	30	17	
Without	76	34	42	
TNM stage				0.030
I-II	82	37	45	
III	41	27	14	
Family history				0.167
With	65	30	35	
Without	58	34	24	

### The prognostic value of USP30-AS1 in colon cancer

**Fig. 3:**
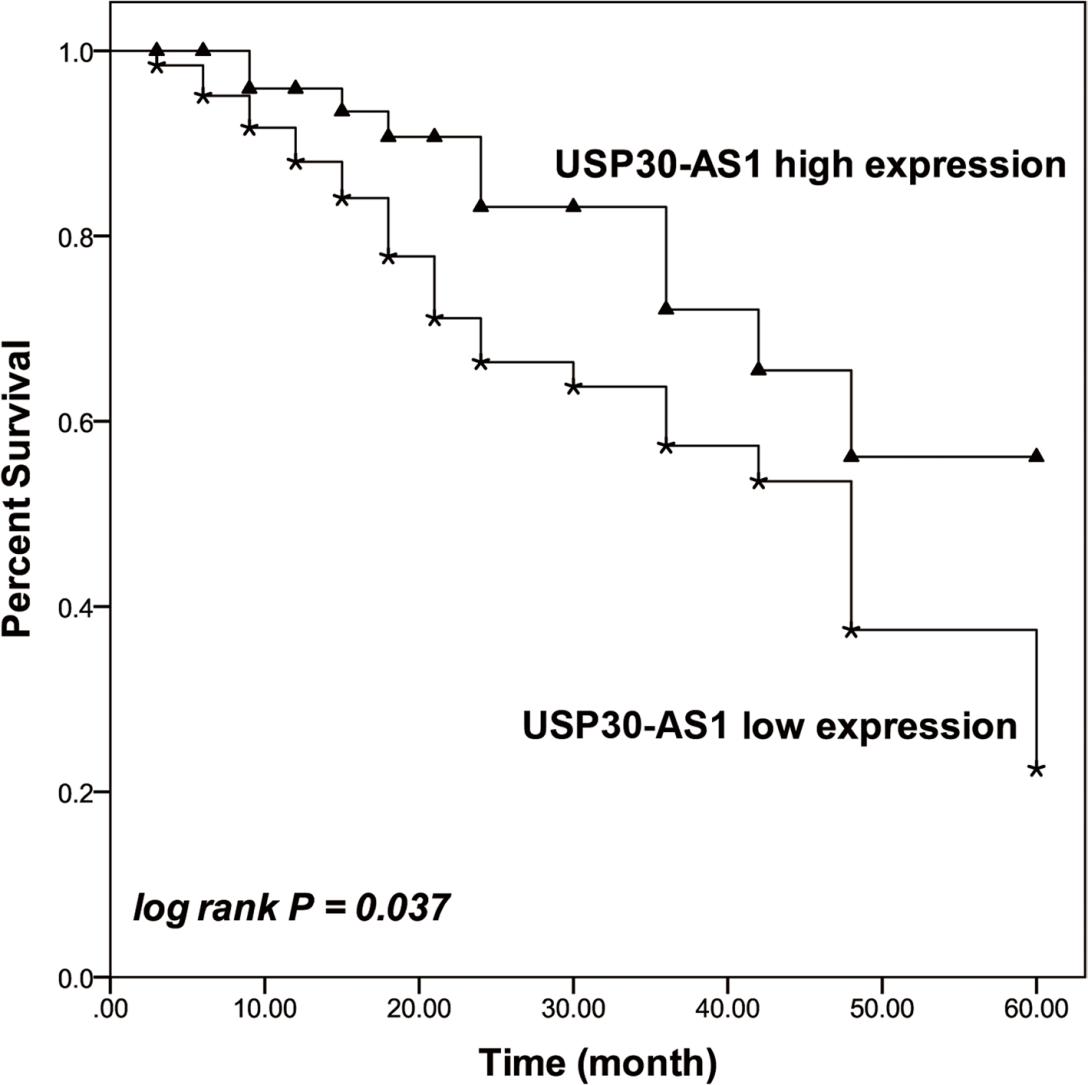
The Kaplan-Meier curve of colon cancer patients based on the expression of USP30-AS1 in collected tissues. The low USP30-AS1 expression was associated with the worse survival rate of patients. log rank *P* = 0.037

The overall survival of patients with low USP30-AS1 expression was found to be dramatically lower than that of patients with high USP30-AS1 expression (log rank *P* = 0.037, Fig. 3). Additionally, USP30-AS1 (95% CI = 1.089–5.182, *P* = 0.030) and TNM stage (95% CI = 1.012–3.973, *P* = 0.046) were identified as independent prognostic factors of colon cancer according to Cox regression analysis (Table 2).

**Table 2:** Cox regression analysis evaluate the prognostic value of patients’ clinical features

	95% CI	HR	*P*
USP30-AS1	1.089–5.182	2.375	0.030
Age	0.759–3.125	1.540	0.231
sex	0.673–2.977	1.415	0.360
Tumor size	0.863–4.061	1.872	0.113
Grade of differentiation	0.889–4.077	1.904	0.097
Lymph node metastasis	0.822–4.547	1.934	0.131
TNM stage	1.012–3.973	2.006	0.046
Family history	0.851–3.692	1.773	0.126

### The effect of USP30-AS1 on the cellular processes of colon cancer and its mechanism

**Fig. 4:**
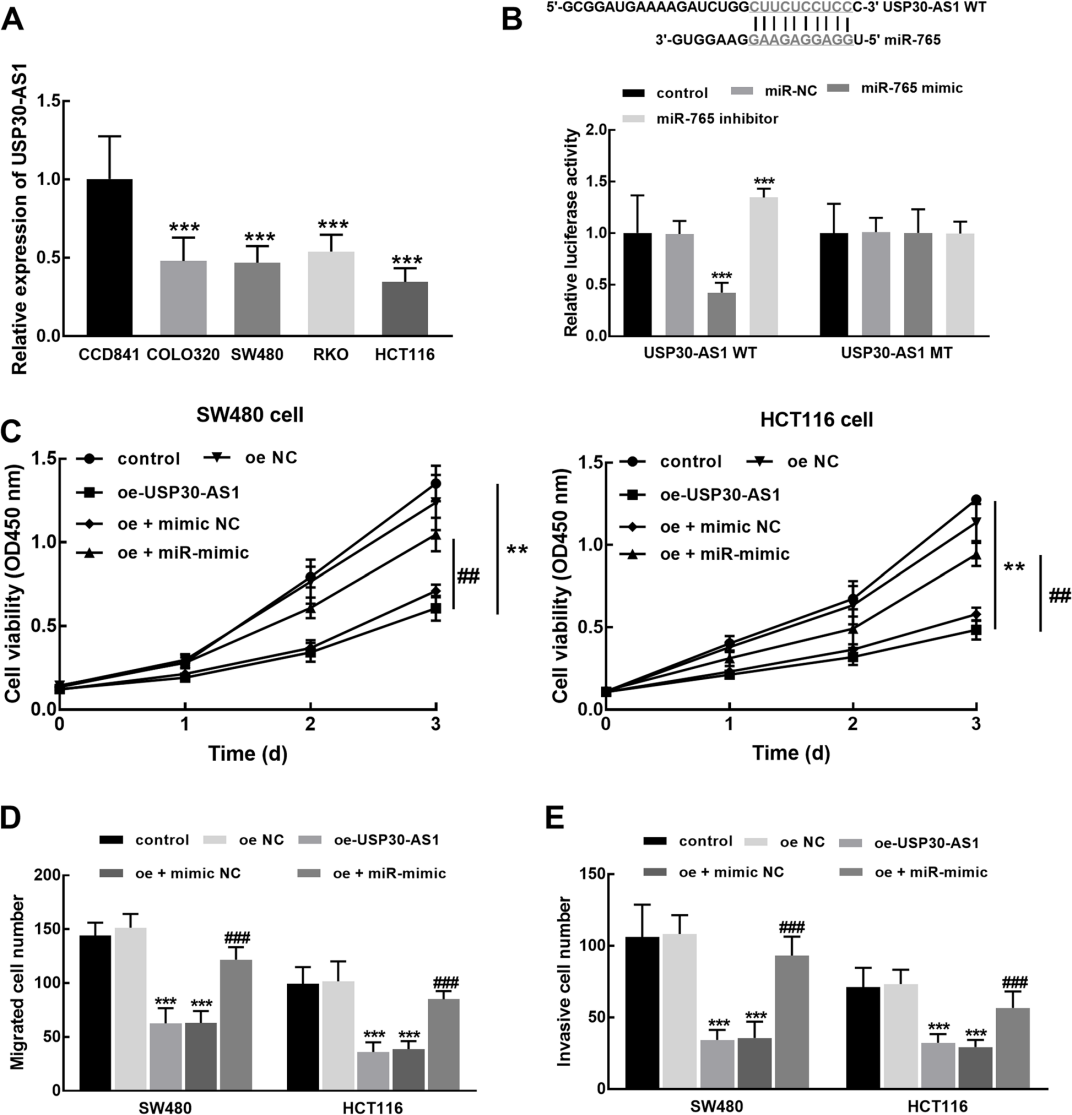
The function of USP30-AS1 in colon cancer cellular processes and its potential mechanism. **A** USP30-AS1 was significantly downregulated in the colon cancer cells compared with the normal cell. **B** miR-765 overexpression dramatically suppressed the luciferase activity of USP30-AS1 WT, which was boosted by miR-765 knockdown. **C–E** The overexpression of USP30-AS1 showed a significant inhibitory effect on the proliferation (**C**), migration (**D**), and invasion (**E**) of SW480 and HCT116 cells. **P < 0.01, ***P < 0.001 relative to the control group; ^##^*P* < 0.01, ^##^*P* < 0.001 relative to the oe-USP30-AS1 group

In colon cancer cells, including COLO320, SW480, RKO, and HCT116cells, the significant downregulation of USP30-AS1 was also observed relative to its expression in the normal CCD841 cell (*P* <0.001, Fig. 4A). While the binding sites between USP30-AS1 and miR-765 were predicted, and the inhibitory effect of miR-765 on the luciferase activity of wild-type USP30-AS1 was disclosed, which was enhanced by the knockdown of miR-765 (*P* < 0.001, Fig. 4B).

In the SW480 and HCT116 cells, two cells sensitive to the downregulation of USP30-AS1, the proliferation of these two cells was found to be dramatically suppressed by the overexpression of USP30-AS1 (*P* < 0.01, Fig. 4C). While the elevated expression of miR-765 could attenuate the inhibitory effect of USP30-AS1 on the proliferation of colon cancer cells (*P* < 0.01, Fig. 4C). Alike, the suppression by USP30-AS1 overexpression was also observed in the migration (Fig. 4D) and invasion (Fig. 4E) of SW480 and HCT116 cells, which was reversed by the overexpression of miR-765 (*P* < 0.001).

## Discussion

With increasing interests in lncRNAs’ function in the development of human disease, a huge number of studies have established corresponding ceRNA networks in colon cancer and dug out differently expressed genes, including lncRNAs, correlated with the clinical prognosis and disease development [13–16]. The abnormal expression of USP30-AS1 has been widely reported, such as in cervical cancer, glioblastoma, ovarian cancer, bladder urothelial carcinoma, and cutaneous melanoma [17–21]. The dysregulation of USP30-AS1 was observed in a lncRNA expression profile of colorectal cancer [8]. Meanwhile, in a previous ceRNA network of colon adenocarcinoma, USP30-AS1 was identified as a signature lncRNA and was speculated to possess a great potential of predicting patients’ recurrence and prognosis [22]. Here, the downregulation of USP30-AS1 in colon cancer was unearthed. The downregulation of USP30-AS1 was associated with the larger tumor size, positive lymph node metastasis, and advanced TNM stage of colon cancer patients, which indicates the severe and malignant progression of patients [23]. Moreover, USP30-AS1 was also evidenced to act as an independent prognostic indicator of colon cancer that predicted a shorter survival time and poorer survival rate of patients, as well the TNM stage. These results suggested the significance of USP30-AS1 in the progression and prognosis of colon cancer.

Meanwhile, the functional roles of USP30-AS1 in tumor cellular processes have also been revealed. In glioblastoma, USP30-AS1 could negatively regulate mitophagy, which might lead to the loss of mitochondrial homeostasis and regulate the tumor progression [10]. Through regulating USP30 and ANKRD13A, USP30-AS1 suppressed the apoptosis of acute myeloid leukemia cells, and therefore improved cell viability [11]. The inhibitory effect of USP30-AS1 on cell growth and metastasis of colon cancer was observed in the present study, suggesting its tumor inhibitor role in colon cancer development. Meanwhile, the downstream mechanism of USP30-AS1 was also disclosed. lncRNAs always served as the sponge of miRNAs, by which lncRNA modulated tumor progression of various cancers. For example, USP30-AS1 could regulate the miR-299-3p/PTP4A1 axis resulting in enhancement of cervical cancer progression [9]. miR-765 was predicted as a potential target of USP30-AS1, and the function of miR-765 has been demonstrated in numerous human cancers. The upregulation of miR-765 in non-small cell lung cancer and esophageal squamous cell carcinoma was indicated to promote disease development and predict patients’ poor outcomes [24, 25]. Herein, the upregulation of miR-765 in colon cancer tissues was observed, which showed a negative correlation with USP30-AS1. While miR-765 showed a negatively regulatory effect on the luciferase activity of USP30-AS1 and alleviated the tumor suppressed the effect of USP30-AS1. Therefore, sponging miR-765 was hypothesized as the molecular mechanism underlying the suppressor role of USP30-AS1 in colon cancer.

However, the deeper mechanism has not been disclosed. The downstream target genes of miR-765 could mediate its function in suppressing or accelerating tumor progression. For instance, INPP4B was suggested as a direct target of miR-765, through which miR-765 promoted hepatocellular carcinoma cell proliferation and enhanced their tumorigenicity [26]. The regulatory effect of miR-765 on the function of lncRNA LINC00511 in the tongue squamous cell carcinoma was demonstrated to be mediated by LAMC2 [27]. Hence, the regulation of target genes by miR-765 is also a potential molecular mechanism involved in the function of USP30-AS1 in colon cancer development.

## Conclusions

In conclusion, downregulated USP30-AS1 could predict the malignant progression and poor prognosis of colon cancer patients. USP30-AS1 could suppress cell proliferation and metastasis of colon cancer via sponging miR-765. The obtained findings provided a reference for the therapeutic strategies of colon cancer.

## Data Availability

Available from the corresponding author.
